# Effectiveness of adjuvant chemotherapy for elderly patients with triple-negative breast cancer

**DOI:** 10.17305/bjbms.2022.8163

**Published:** 2023-05-01

**Authors:** Qiusheng Guo, Tian Lan, Yunyan Lu, Zujian Hu, Haibin Xu, Xiaojia Wang, Xiying Shao, Xueyan Fu

**Affiliations:** 1 Department of Medical Oncology, Affiliated Jinhua Hospital, Zhejiang University School of Medicine, Jinhua, China; 2 Department of Breast Surgery, Hangzhou TCM Hospital Affiliated to Zhejiang Chinese Medical University, Hangzhou Hospital of Traditional Chinese Medicine, Hangzhou, China; 3 Department of Cardiology, The First People’s Hospital of Xiaoshan District, Xiaoshan Affiliated Hospital of Wenzhou Medical University, Hangzhou, China; 4 Department of Medical Oncology (Breast Cancer), Cancer Hospital of the University of Chinese Academy of Sciences/Zhejiang Cancer Hospital, Hangzhou, China; 5 Institute of Cancer and Basic Medicine (IBMC), Chinese Academy of Sciences, Hangzhou, China

**Keywords:** Adjuvant chemotherapy, observation, triple-negative breast cancer (TNBC), geriatric oncology

## Abstract

There is little evidence determining whether elderly patients (from 70 to 90 years old) with triple-negative breast cancer (TNBC) could benefit from adjuvant chemotherapy (AC). This study explores the effect of AC in these population following surgery. A total of 4610 patients were identified in the Surveillance, Epidemiology, and End Results database (2010–2018). Multiple imputation by chained equations was performed to impute missing data. Inverse probability of treatment weighting (IPTW) was applied to reduce the selection bias. IPTW-adjusted Kaplan–Meiers survival analysis and Cox proportional hazards models were performed to compare breast cancer-specific survival (BCSS) and overall survival (OS) in the two treatment groups. The patients were classified into the chemotherapy (*n* ═ 1989) and the observation (*n* ═ 2621) groups. The percentage of patients receiving AC vs observation increased significantly from 2010 to 2018 (estimated annual percentage change, 1.49%; 95%CI, 0.75–2.16%, *p* ═ 0.002). The 5-year IPTW-adjusted rates of BCSS and OS in the AC group were better than that in the observation group (BCSS: 82.32% vs 78.42%, *p* ═ 0.010; OS: 75.54% vs 64.65%, *p* < 0.001). The patients could benefit from AC based on the results of IPTW-adjusted Cox proportional hazards regression analysis (BCSS: HR, 0.77, 95%CI, 0.62–0.94, *p* ═ 0.012; OS: HR, 0.66, 95%CI, 0.57–0.78, *p* < 0.001). AC was associated with a significant outcome benefit across the year at diagnosis, marital status, stage, lymph node, surgery, and radiation subgroups (all *p* < 0.050). Patients with T1ab could not benefit from AC (*p* > 0.050). In conclusion, we presented a BCSS and OS benefit from AC in elderly patients with TNBC. AC remained a reasonable treatment approach in these specific patients. For the patients with T1ab, de-escalated treatment would be administrated with caution.

## Introduction

Breast cancer is the most common female malignancy worldwide [1]. Based on the expression of human epidermal growth factor 2 (HER2), estrogen receptor (ER), progesterone receptor (PR), and Ki67, physicians classified breast cancer as luminal A, luminal B, Her2-enriched, and triple-negative subtype [2]. The triple-negative breast cancer (TNBC) accounts for approximately 20% of breast cancer cases and lacks the expression of ER, PR, and HER2. TNBC usually appears at a higher rate of local recurrences and distant metastases and presents a worse prognosis than other breast cancer subtypes [3]. Although some targeted therapies were investigated in clinical trials on advanced TNBC, chemotherapy remained the backbone of adjuvant treatment against early TNBC [4–7].

The global population is aging at a sharp rate. By 2030, 20% of all adults are estimated to be 65 and above [8]. Aging is the top risk factor for cancer. The incidence of breast cancer in older adults will increase by 67% in 2030 [9]. However, the evidence-based treatment decisions for these elderly patients with TNBC were inadequate because less than 20% of these patients were included in the current clinical trials [10]. In the CALGB 49907, which enrolled women aged over 65 with early breast cancer, standard adjuvant chemotherapy (AC) (doxorubicin and cyclophosphamide or cyclophosphamide, methotrexate, and fluorouracil) showed significant improvements in breast cancer-specific survival (BCSS), but not in overall survival (OS) compared with capecitabine [11]. There were remarkable differences in response to cancer treatments, notably between older people and younger adults, due to age-associated organ function, comorbidities, life expectancy, and chemotherapy tolerance. Nevertheless, physicians are forced to extrapolate from the clinical trials conducted in healthier and younger patients when making treatment decisions [12].

There are few population-based retrospective studies evaluating the impact of AC in older women with TNBC. One study was derived from the National Cancer Database, and the other was derived from the Swedish National Breast Cancer Register [13, 14]. These studies presented a significant prognostic benefit from AC for elderly TNBC patients. In clinical practice, clinicians’ decision making may be prone to undertreatment for older patients due to the increased severity of the side effects caused by chemotherapy [15, 16]. Considering the increasing number of cases of older TNBC patients and the limited evidence of clinical trials, it is of immense importance to assess the effectiveness of chemotherapy in older patients with TNBC using the population-based registry study. Therefore, we conducted a retrospective study using the Surveillance, Epidemiology, and End Results (SEER) database in order to investigate the benefits of AC in older women with TNBC in the present study.

## Materials and methods

### Data source and patient population

TNBC data were extracted by using the SEER-stat software (SEER*Stat 8.3.9) from the SEER 18 regions database [Incidence-SEER 18 Research Plus Data, 18 Registries, Nov 2020 Sub (2000–2018)] that covers approximately 34.6% of the U.S. population [17]. We could access the SEER database with the permission ID number (19574-Nov2020). This study was exempted from the Ethics Committee of Hangzhou Hospital of Traditional Chinese Medicine as hospital, physician, and patient information were deidentified. Since the information on Her2 was unavailable before 2010, we included patients diagnosed between 2010 and 2018. The inclusive criteria to identify eligible patients were set as follows: (1) female patients; (2) patients aged 70–89 years at diagnosis; (3) histology ICD-O-3 (International Classification of Diseases for Oncology, 3rd edition) restricted to infiltrating ductal breast cancer (8500); (4) TNBC; (5) patients treated with surgery (breast-conserving surgery or mastectomy); (6) survival times ≥ 1 month. The patients treated with breast-conserving surgery who did not receive radiation were excluded from the present study. Then the eligible patients were divided into the observation group and the AC group. The primary and secondary outcome measures were BCSS and OS, respectively.

### Study variables

The SEER database provided the following clinicopathological factors: patient-associated demographics, clinicopathologic features, treatment-related variables, and prognostic information. Patient-associated demographics included age (70–79 or 80–89), year at diagnosis (2010–2014 or 2015–2018), marital status (married or unmarried), race (white, black, or others), and median household income (<$40,000, $40,000–$49,999, $50,000–$59,999, $60,000–$69,999, or >$70,000). Tumor-related information was grade, tumor size, lymph node status, and TNM stage. Treatment data consisted of surgery (breast-conserving surgery or mastectomy) and radiation.

### Statistical analysis

Multiple imputations by chained equations were performed using the mice R package to impute missing data for the grade (14.3% missing), lymph node status (6.1% missing), marital status (5.0% missing), tumor size (1.4% missing), stage (0.7% missing), and race (0.4% missing), with the assumption that the missing data were missing at random [18]. According to the rule of thumb that the number of imputations should be similar to the percentage of incomplete cases [19], the number of imputations in this study was set as 30. All variables used in the subsequent analysis were included in the imputation model [20].

To reduce the selection bias in this retrospective and non-random study, the observed differences in baseline features between the two treatment groups were controlled with the inverse probability of treatment weighting (IPTW) method. IPTW had superior performance over propensity score matching, such as retaining all the cases in the subsequent analysis [21].

A multivariate logistic regression was used to calculate propensity scores, the probability of receiving a treatment (chemotherapy or observation in this study), in each imputed dataset.

The independent variables in this model were year at diagnosis, age, marital status, median household income, race, grade, tumor size, lymph node status, surgery, and radiation. Then Rubin’s rules were applied to combine the estimated propensity scores from the 30 imputed datasets. Standardized mean difference (SMD) of less than 0.1 is considered as a good match of covariates between two treatment groups [22].

Adjusted survival analysis was conducted using the Kaplan–Meier curves and the Log-rank test based on IPTW [23]. We applied univariable Cox proportional hazards regression analysis to estimate the IPTW-adjusted hazard ratio (HR) of AC vs observation [24]. Similarly, in the subgroup analyses based on year at diagnosis, age, marital status, race, stage, tumor size, lymph node, surgery, and radiation, we further identified the sensitive population via the IPTW-adjusted HR of AC vs observation. The post-weighting balance was observed in all subgroups.

At last, we reported the E-value by performing additional sensitivity analyses to estimate the assessment of robustness to biases, such as unmeasured or uncontrolled confounding. A high E-value revealed that the strong confounder associations would be needed to explain away the observed treatment-outcome relationship. Two-sided statistical significance was set as *p* < 0.050. All statistical analyses were performed using the R statistical software v4.1.2.

**Figure 1.**
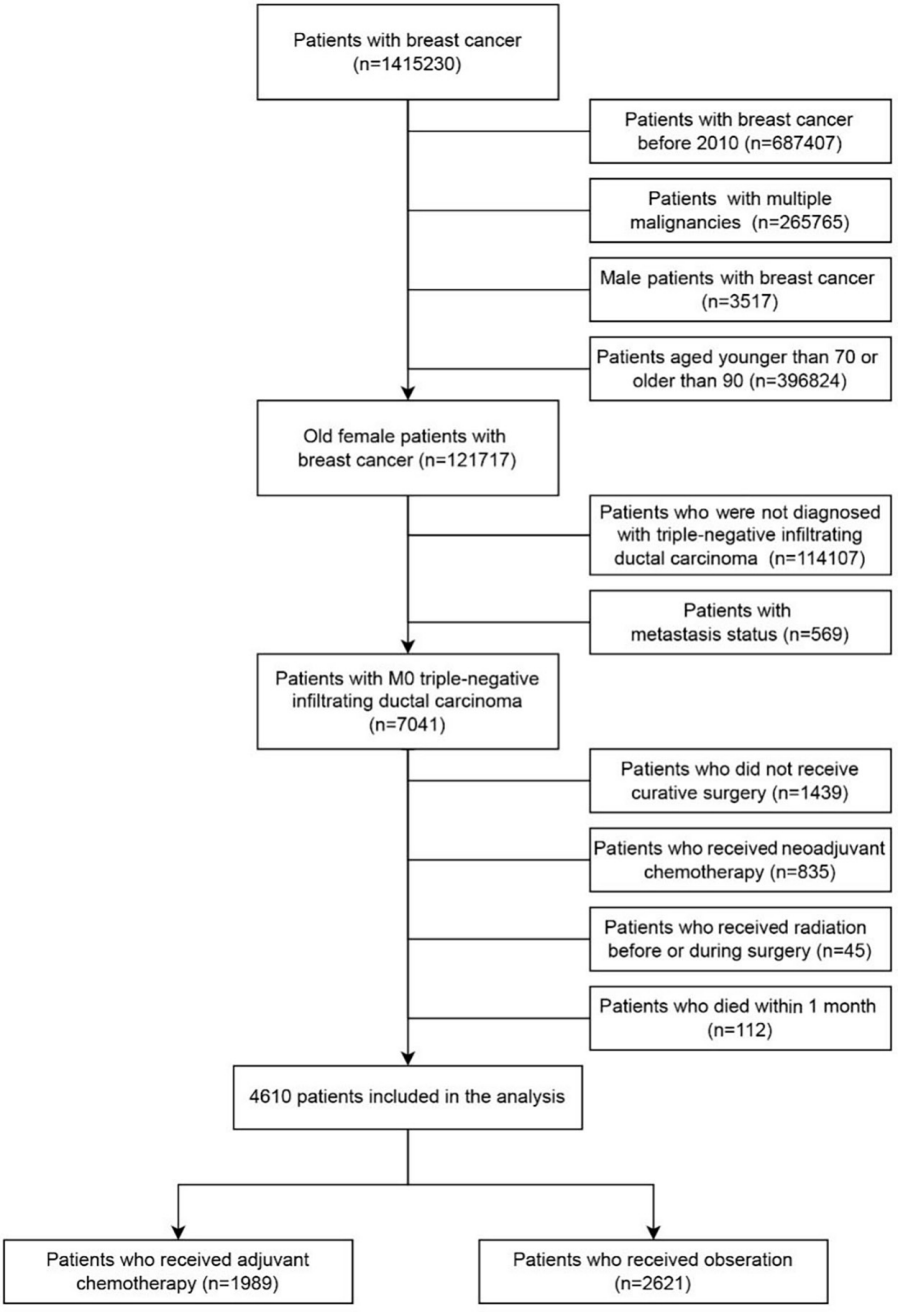
Flowchart for the selection of older patients with triple-negative breast cancer (TNBC) after surgery in the SEER database (2010–2018).

## Results

### Unweighted and weighted baseline patient features

**Figure 2.**
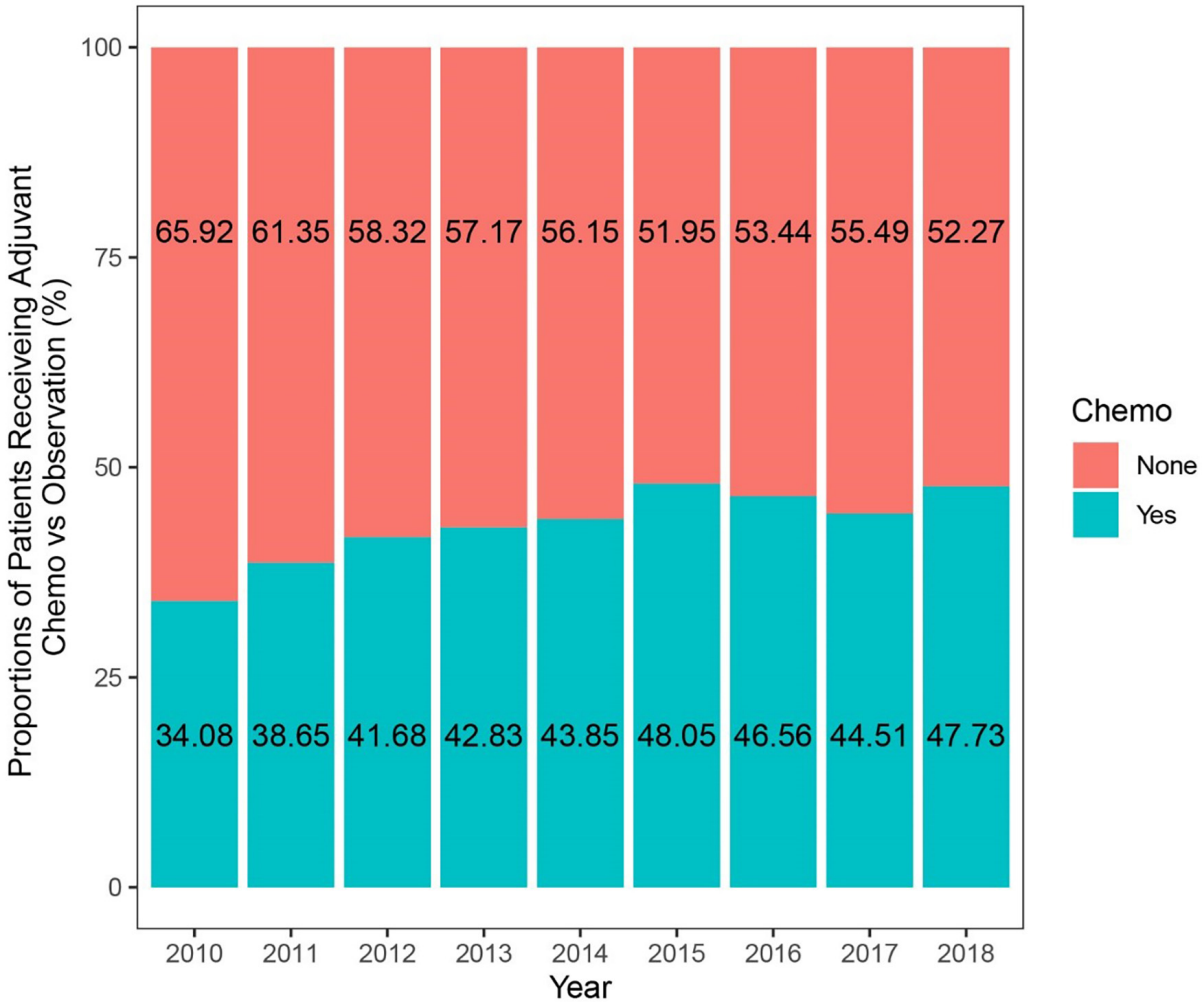
Adoption rates of adjuvant chemotherapy for old patients with triple-negative breast cancer (TNBC) in the unweighted study population.

**Figure S1.**
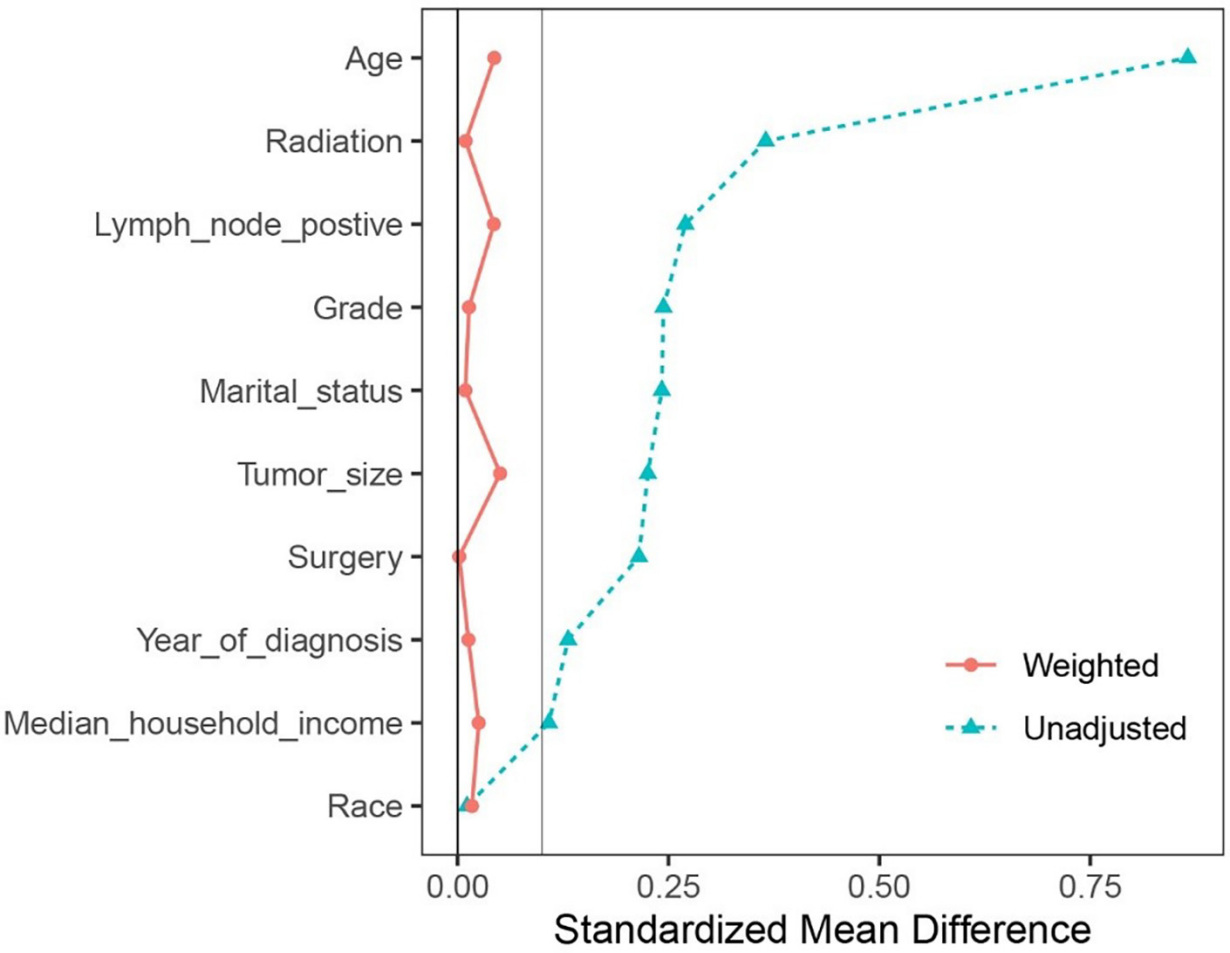
Standardized mean differences of the cohorts before and after inverse probability of treatment weighting.

A total of 1,415,230 patients with breast cancer were extracted from the SEER database between 2010 and 2018. We identified 4610 elderly female patients with nonmetastatic TNBC who received surgery according to the inclusion and exclusion criteria, of whom 1989 were treated with AC and 2621 with observation (Figure 1). The comparison of the unweighted and weighted differences between the two groups is shown in Table 1. In the unweighted cohort, there were significant differences among most demographic, socioeconomic, and clinical parameters of interest between the AC and observation groups. The AC group presented with a higher portion of lower age, married status, higher grade, larger tumor size, and more lymph node metastasis compared to the observation group. The portion of married patients in the AC group was higher than that in the observation group. Married status could contribute to stronger financial resources, ultimately affecting access to sufficient treatment [25]. Meanwhile, the patients in the AC group were more prone to receive breast-conserving surgery and radiation. The percentage of patients receiving AC vs observation increased significantly from 2010 to 2018 (estimated annual percentage change, 1.49%; 95%CI, 0.75%–2.16%, *p* ═ 0.002; Figure 2). After IPTW adjustment, it indicated that the two groups in the weighted cohort were comparable because the standardized differences of all features were less than 0.1 (Figure S1).

**Table 1:** Baseline characteristics of old patients who received adjuvant chemotherapy vs observation after operation for triple-negative breast cancer in unweighted and weighted study populations

	**Unweighted Study Population, No. (%)**			**Weighted Study Population, No. (%)**		
**Characteristics**	**Observation *n* ═ 2688**	**Adjuvant Chemotherapy *n* ═ 2034**	***P* value**	**Observation**	**Adjuvant Chemotherapy**	***P* value**
*Year of diagnosis*			<0.001			0.727
2010–2014	1533 (58.5)	1034 (52.0)		1444.0 (55.0)	1061.3 (54.3)	
2015–2018	1088 (41.5)	955 (48.0)	<0.001	1182.1 (45.0)	891.6 (45.7)	0.308
*Age, years; mean (SD)*	78.53 (5.34)	74.47 (3.95)		76.72 (5.31)	76.50 (4.88)	
*Marital status*			<0.001			0.807
Unmarried	1478 (59.7)	905 (47.7)		1339.0 (54.1)	999.6 (53.7)	
Married	998 (40.3)	992 (52.3)		1134.5 (45.9)	862.9 (46.3)	
*Median household income, $*			0.010			0.977
<40,000	121 (4.6)	110 (5.5)		133.7 (5.1)	100.2 (5.1)	
40,000–49,999	365 (13.9)	280 (14.1)		381.5 (14.5)	275.0 (14.1)	
50,000–59,999	454 (17.3)	322 (16.2)		441.5 (16.8)	315.6 (16.2)	
60,000–69,999	786 (30.0)	521 (26.2)		736.0 (28.0)	550.9 (28.2)	
>70,000	895 (34.1)	756 (38.0)		933.3 (35.5)	711.3 (36.4)	
*Race*			0.931			0.901
White	2011 (77.0)	1515 (76.6)		2001.8 (76.4)	1475.8 (75.9)	
Black	404 (15.5)	314 (15.9)		420.5 (16.1)	315.1 (16.2)	
Others	198 (7.6)	150 (7.6)		196.2 (7.5)	154.0 (7.9)	
*Grade*			<0.001			0.733
I+II	686 (29.7)	331 (19.3)		571.2 (24.9)	413.1 (24.3)	
III	1623 (70.3)	1385 (80.7)		1720.4 (75.1)	1283.9 (75.7)	
*Tumor size*			<0.001			0.449
T1	1513 (58.8)	997 (50.6)		1413.7 (54.8)	1071.6 (55.3)	
T2	868 (33.7)	875 (44.4)		1000.4 (38.8)	721.7 (37.2)	
T3	191 (7.4)	100 (5.1)		164.4 (6.4)	145.6 (7.5)	
*Lymph node*			<0.001			0.77
N0	1910 (79.3)	1316 (67.8)		1825.2 (74.5)	1378.2 (72.8)	
N1	331 (13.7)	409 (21.1)		408.5 (16.7)	343.1 (18.1)	
N2	97 (4.0)	147 (7.6)		127.5 (5.2)	105.1 (5.6)	
N3	72 (3.0)	70 (3.6)		89.1 (3.6)	66.8 (3.5)	
*Surgery*			<0.001			0.958
BCS	1312 (50.1)	1207 (60.7)		1420.7 (54.1)	1058.5 (54.2)	
Mastectomy	1309 (49.9)	782 (39.3)		1205.3 (45.9)	894.4 (45.8)	
*Radiation*			<0.001			0.803
None	1144 (43.6)	527 (26.5)		980.2 (37.3)	719.9 (36.9)	
Yes	1477 (56.4)	1462 (73.5)		1645.9 (62.7)	1233.0 (63.1)	

BCS: Breast-conserving surgery.

The results of multivariable logistic regression analysis predicting receipt of AC vs observation in the unweighted cohort are shown in Table 2. Patients who were married (compared to unmarried), had higher grade, lymph node metastasis, or underwent radiation were more likely to receive adjuvant treatment.

### Adjuvant chemotherapy vs observation

**Figure 3.**
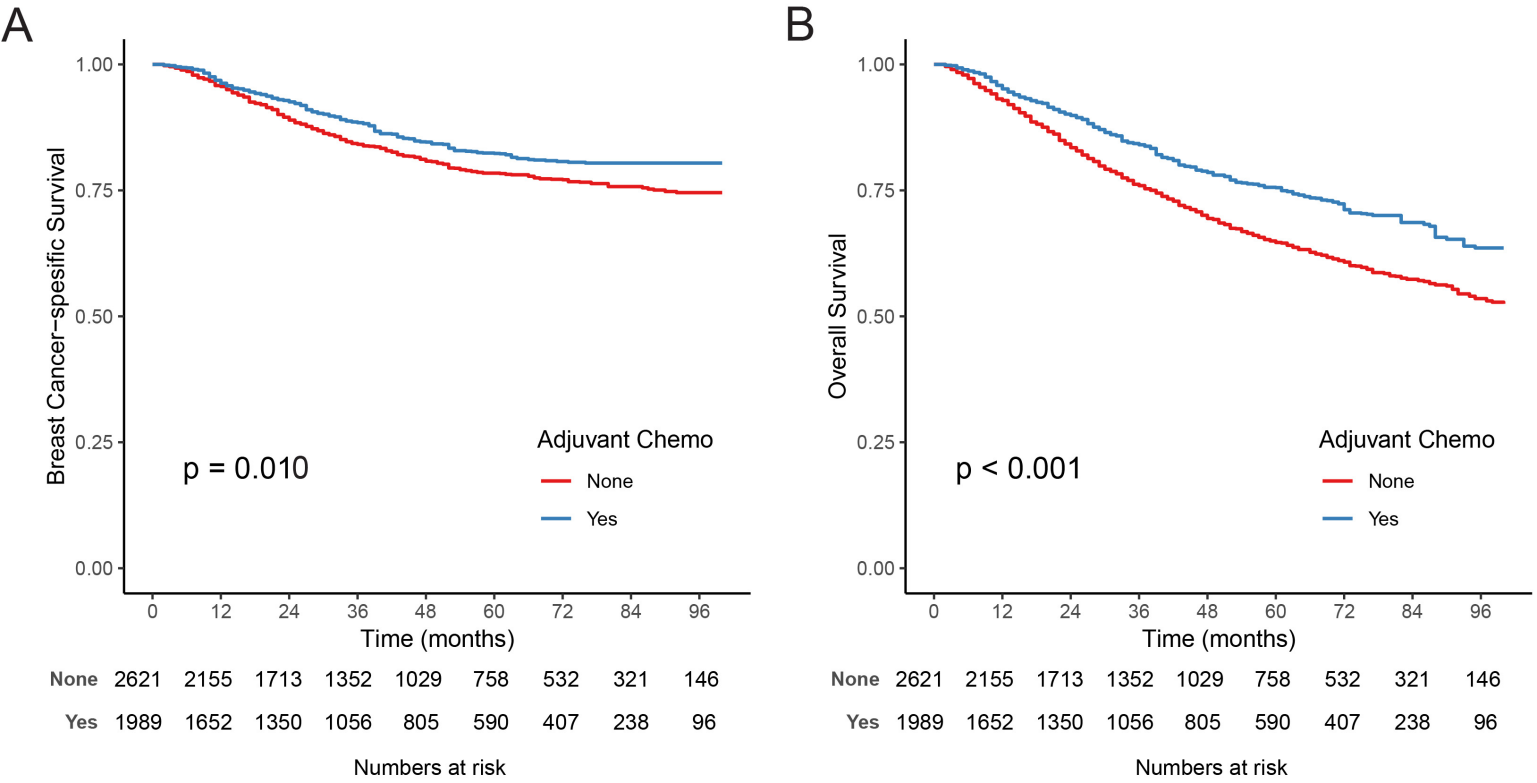
I**PTW-adjusted estimates of breast cancer-specific survival (A) and overall survival (B) based on receipt of adjuvant chemotherapy for old patients with triple-negative breast cancer.** IPTW: Inverse probability of treatment weighting.

In the weighted cohort, the median follow-up was 38 months, and the interquartile range was 48 months (17–65). The IPTW-adjusted Kaplan–Meier analysis confirmed that BCSS in the AC group was longer than in the observation group (*p* ═ 0.010; Figure 3A). The 5-year IPTW-adjusted rates of BCSS were estimated as 82.32% and 78.42% for the AC and the observation groups, respectively. Furthermore, the results of IPTW-adjusted Kaplan–Meier analysis demonstrated that 5-year OS rates for AC vs observation were 75.54% and 64.65%, respectively (*p* < 0.001; Figure 3B). In IPTW-adjusted Cox proportional hazards regression analysis, elderly patients could benefit from AC (HR, 0.77, 95%CI, 0.62–0.94, *p* ═ 0.012 for BCSS; HR, 0.66, 95%CI, 0.57–0.78, *p* < 0.001, for OS).

### Subgroup analysis

**Figure 4.**
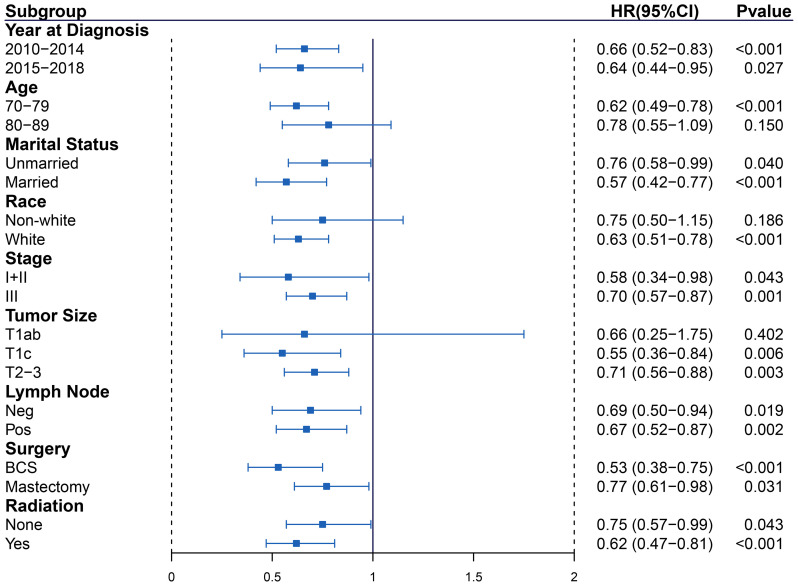
**Forest plot depicting IPTW-adjusted hazard ratios (breast cancer-specific survival) of adjuvant chemotherapy to observation in subgroups.** IPTW: Inverse probability of treatment weighting; HR: Hazard ratio; CI: Confidence interval; BCS: Breast-conserving surgery.

**Figure S2.**
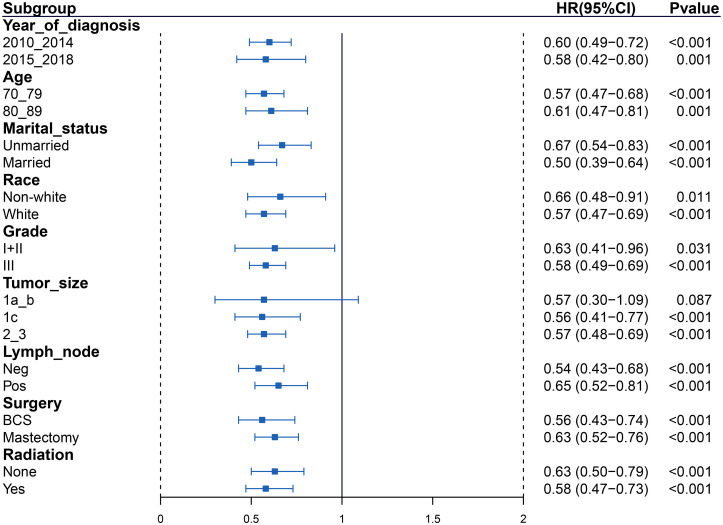
**Forest plot depicting the association between adjuvant chemotherapy and IPTW-adjusted overall survival in subgroups.** HR: Hazard ratio; CI: Confidence interval; IPTW: Inverse probability of treatment weighting; BCS: Breast-conserving surgery.

Subgroup analyses were performed to further explore the IPTW-adjusted HRs of AC vs observation outcome based on year at diagnosis, age, marital status, race, stage, tumor size, lymph node, surgery, and radiation (Figure 4 for BCSS, Figure S2 for OS). AC was related to a considerable outcome benefit across years at diagnosis, marital status, stage, lymph node, surgery, and radiation subgroups (all *p* < 0.050). However, patients with T1ab could not benefit from AC.

### Sensitivity analyses

In the complete case analysis, the 5-year IPTW-adjusted rates of prognosis in the AC group were better than that in the observation group (BCSS: 81.75% vs 78.24%, *p* ═ 0.036; OS: 75.31% vs 65.19%, *p* < 0.001). AC was related to a better outcome based on the IPTW-adjusted Cox proportional hazards regression analysis (HR, 0.79, 95%CI, 0.63–0.98, *p* ═ 0.039 for BCSS; HR, 0.69, 95%CI, 0.58–0.82, *p* < 0.001 for OS).

The E-value for an estimate is 1.92, indicating that the observed HR of 0.77 could be explained away by an unmeasured confounder that was associated with both AC and BCSS by a risk ratio of 1.92-fold each, above and beyond the measured confounders, but weaker confounding could not do so. The unmeasured confounder would not suffice to explain away the AC effect estimate. In addition, the E-value for OS HR is 2.40.

## Discussion

The findings of our study demonstrated that AC was related to improved BCSS and OS in older patients with nonmetastatic TNBC. It was interesting that chemotherapy improved OS, but not BCSS, in patients aged over 80 years. Less intensive chemotherapy should be applied with caution for patients aged over 80 years based on contradictory results. According to the present National Comprehensive Cancer Network guideline for patients under 70 years, chemotherapy should be considered for T1bN0M0 TNBC and exempt for T1aN0M0 TNBC. But our study presented that older patients with T1abN0M0 TNBC could not get survival benefits (BCSS and OS) from chemotherapy. De-escalation of chemotherapy may be an acceptable approach for these specific patients.

Clinicians should consider the physical status of patients, like function, falls, comorbidity, cognition, depression, and nutrition, when recommending chemotherapy for older TNBC women in terms of the American Society of Clinical Oncology Guideline [26]. Some clinical tools, including cancer and aging research group or chemotherapy risk assessment scale for high-age patient’s tools, instrumental activities of daily living, mini-mental state examination, mini nutritional assessment, and geriatric depression scale, were helpful for clinicians in making geriatric assessments and sharing a decision-making process with older patients [26].

**Table 2:** Multivariable logistic regression model predicting receipt of adjuvant chemotherapy vs observation in the unweighted study population

**Characteristics**	**OR (95% CI)**	***P* value**
*Year of diagnosis*		
2010–2014	1.00 (Reference)	
2015–2018	1.25 (1.09–1.43)	<0.001
Age, mean (SD)	0.82 (0.80–0.83)	<0.001
*Marital status*		
Unmarried	1.00 (Reference)	
Married	1.22 (1.06–1.41)	0.006
*Median household income, $*		
<40,000	1.00 (Reference)	
40,000–49,999	0.75 (0.53–1.06)	0.106
50,000–59,999	0.61 (0.43–0.85)	0.004
60,000–69,999	0.60 (0.43–0.83)	0.002
>70,000	0.83 (0.60–1.15)	0.272
*Race*		
White	1.00 (Reference)	
Black	0.88 (0.73–1.07)	0.194
Others	1.04 (0.80–1.35)	0.792
*Grade*		
I+II	1.00 (Reference)	
III	1.85 (1.56–2.19)	<0.001
*Tumor size*		
T1	1.00 (Reference)	
T2	2.00 (1.71–2.34)	<0.001
T3	0.96 (0.69–1.33)	0.785
*Lymph node*		
N0	1.00 (Reference)	
N1	2.43 (2.00–2.97)	<0.001
N2	3.03 (2.15–4.27)	<0.001
N3	2.51 (1.64–3.84)	<0.001
*Surgery*		
BCS	1.00 (Reference)	
Mastectomy	1.12 (0.84–1.49)	0.453
*Radiation*		
None	1.00 (Reference)	
Yes	2.39 (1.81–3.17)	<0.001

BCS: Breast-conserving surgery.

It remains controversial whether AC could provide a survival benefit in older patients with TNBC. Some studies conducted before 2000 presented that chemotherapy could decrease all cause mortality in lymph node-positive, ER-negative, older patients [27, 28]. Two recent studies, one based on the Swedish National Breast Cancer Register, and the other from the National Cancer Database, suggested that chemotherapy yielded prognostic benefits for older TNBC women [13, 14]. On the contrary, several authors reported that the TNBC in older patients may be an indolent disease, and old patients may be insensitive to chemotherapy [29, 30]. Despite less aggressive therapy, the older TNBC patients had equivalent outcomes to younger patients [31].

There were several studies associated with the SEER database focusing on elderly TNBC patients. Retrospective research based on the SEER database indicated a prognostic difference between patients aged 18–69 with TNBC and patients aged 70 or older, which may be caused by undertreatment [32]. Another study derived from the SEER database (1992–1999) revealed an improved survival from AC in older women with hormone receptor-negative breast cancer [28]. Chemotherapy was associated with a significant reduction in breast cancer death among patients over 65 years with ER-negative and lymph node-positive breast cancer, according to the SEER database (1991–1999) [27]. The chemotherapy regimens have changed over the years. The adjuvant treatment in the 1990s (usually cyclophosphamide, methotrexate, and fluorouracil) was different from that in the 2010s (anthracycline-based or taxane-based chemotherapy regimens). Therefore, it is essential to conduct a population-based study based on the SEER database (2010–2018). To our knowledge, the current research is the first study derived from the latest SEER database to provide extraordinary evidence supporting AC for old patients with TNBC via the implementation of multiple imputation and IPTW.

Our study should be interpreted with the following limitations. First, some significant factors associated with clinical and geriatric assessment, including Ki67, the Charlson–Deyo index, and BRCA1/2, were unavailable in the SEER database. The regimen changes during 2010–2018, and the lack of detailed chemotherapy regimens information may lead to bias. Residual unmeasured covariates may have impacted the founding of our study. In the current study, the results of a sensitivity analysis revealed a moderately robust outcome that an unmeasured confounder should have at least a 1.92-fold stronger association with both chemotherapy and prognosis in comparison with the relationship between chemotherapy and survival. Second, despite the implementation of some statistical approaches, selection bias was inevitable due to the retrospective nature of this study. Although the difficult recruitment of these specific patients, further prospective randomized control trials should be warranted.

## Conclusion

In conclusion, the study presented a BCSS and OS benefit from AC in old patients with TNBC. AC should remain a reasonable treatment approach for these specific patients. Clinicians should take into consideration the function, falls, comorbidity, cognition, depression, and nutrition to administrate de-escalated treatment with caution for patients over 80 years or with T1ab.

## Supplemental Data
